# Expression of matrix metalloproteinase 12 is highly specific for non-proliferating invasive trophoblasts in the first trimester and temporally regulated by oxygen-dependent mechanisms including HIF-1A

**DOI:** 10.1007/s00418-017-1608-y

**Published:** 2017-10-09

**Authors:** Ursula Hiden, Christian P. Eyth, Alejandro Majali-Martinez, Gernot Desoye, Carmen Tam-Amersdorfer, Berthold Huppertz, Nassim Ghaffari Tabrizi-Wizsy

**Affiliations:** 1 Department of Obstetrics and Gynecology, Medical University of Graz, Graz, Austria; 2 Institute of Pathophysiology and Immunology, Center of Molecular Medicine, Medical University of Graz, Graz, Austria; 3 Institute of Cell Biology, Histology and Embryology, Medical University of Graz, Graz, Austria

**Keywords:** Matrix metalloproteinase 12, Trophoblast invasion, HIF-1α, Hypoxia

## Abstract

During first trimester pregnancy, trophoblast cells invade from the placenta into the maternal decidua where they anchor the placenta and remodel luminal structures like spiral arteries. This process depends on proteases secreted by invading trophoblasts, which degrade extracellular matrix (ECM). We here aimed to identify proteases particularly important for trophoblast invasion. We generated a list of proteases capable of degrading decidual ECM and trophoblast integrins using MEROPS database and compared expression of these proteases between primary trophoblasts isolated from first trimester placenta (FT, *n* = 3), representing an invasive phenotype, vs trophoblasts isolated from term pregnancy (TT, *n* = 3), representing a non-invasive trophoblast phenotype. Matrix metalloproteinase 12 (*MMP12*) revealed highest expression levels in FT, with absent expression in TT. In situ hybridisation and immunofluorescence localised MMP12 specifically to extravillous trophoblasts (evCT) whilst Ki67 co-staining revealed that proliferating trophoblasts of the cell columns were almost negative for MMP12. Quantification revealed a decline in MMP12 positive evCT at the end of first trimester, when oxygen levels start rising. *MMP12* promoter analysis identified potential binding sites for hypoxia-inducible factor (HIF-1) and other oxygen-sensitive transcription factors. Moreover, MMP12 protein was increased by low oxygen in FT in vitro and by addition of a HIF-1α activator. Collectively, MMP12 is a highly expressed protease specific for invasive evCT during the first trimester. MMP12 down regulation by increasing oxygen concentration enables temporal expression control of MMP12 and involves several mechanisms including HIF-1α. These findings suggest MMP12 involved in trophoblast invasion during the first trimester.

**Electronic supplementary material:**

The online version of this article (doi:10.1007/s00418-017-1608-y) contains supplementary material, which is available to authorized users.

## Introduction

During the first trimester of pregnancy, a specific subset of placental trophoblasts differentiate into invasive, extravillous cytotrophoblasts (evCT) and invade into the maternal decidua. Along their invasion pathway, evCT form compact cell columns that anchor the placenta to the uterine wall. A number of evCT invade further towards luminal structures within the placental bed (Moser and Huppertz 2017), such as the uterine spiral arteries, which are remodelled into low resistance vessels. Trophoblast invasion depends on the function of proteases that are capable to degrade extracellular matrix (ECM) components (Ghaffari-Tabrizi-Wizsy et al. 2014; Cohen et al. 2006; Anacker et al. 2011) present in the decidua, i.e. various collagens, laminin, fibronectin, fibrillin and vitronectin (Kemp et al. 2002; Oefner et al. 2015; Aplin et al. 2000). Furthermore, before evCT can start to move and migrate through the degraded ECM along their invasion pathway, evCT need to detach their connections to the ECM and thus, cleave integrins through which they adhere (Kemp et al. 2002). In fact, various proteases were identified in invading trophoblasts in the first trimester, which participate in the process of invasion. For instance, matrix metalloproteinases (MMPs) are a family of key proteinases involved in ECM degradation, and various MMPs including MMP2, 9, 7 and 14 were implicated in trophoblast invasion in vitro and in vivo (reviewed by (Cohen et al. 2006)). Besides MMPs, also other proteases contribute to ECM degradation or to integrin shedding to eventually facilitate trophoblast invasion, such as members of the ADAMs (a disintegrin and metalloproteinase) family (Pollheimer et al. 2014).

Trophoblast invasion is tightly regulated by multiple factors including cytokines, hormones and growth factors (Bischof and Irminger-Finger 2005). However, oxygen is one of the most potent regulators (Huppertz et al. 2009; Pringle et al. 2010): Whilst a population of invasive trophoblasts modifies and widens the uterine spiral arteries in the first weeks of gestation, some trophoblasts form plugs to prevent immediate maternal blood flow to the placenta, thus creating a low oxygen environment within the placenta. Only at the end of the first trimester do plugs start to detach and placental oxygen levels rise (Kaufmann et al. 2003; Jauniaux et al. 2000). This rise in oxygen is an important signal for foeto-placental development and limits trophoblast invasion. Concomitantly, expression of various proteases involved in the invasion process decreases (Xu et al. 2000).

Our aim was to identify proteases which are particularly important for trophoblast invasion. For this purpose we generated a list of proteases that degrade ECM components present in the decidua and integrins expressed by invasive trophoblasts and compared these proteases between isolated primary trophoblasts from first trimester placenta (FT), representing an invasive, mostly HLA-G positive phenotype (Ghaffari-Tabrizi-Wizsy et al. 2014), and trophoblasts from term placenta (TT), representing a non-invasive trophoblast population (Hohn and Denker 2002; Shimonovitz et al. 1994). Among such potentially invasion-relevant proteases, the protease with highest expression levels in isolated first trimester trophoblasts was *MMP12*, with absent expression in trophoblasts at term. Therefore, we further investigated placental gene and protein expression patterns of MMP12 throughout the first trimester, explored a possible role of oxygen in its regulation and thus, identified MMP12 as a new, spatio-temporally regulated protease expressed by invasive, evCT of the cell columns.

## Materials and methods

### Ethics statement

The study was approved by the Ethics Committee of the Medical University of Graz. All women gave written informed consent for collection and investigational use of tissues.

### Tissue collection

Placental villi and decidua from first trimester were obtained from elective vaginal terminations of first-trimester pregnancies (gestational age 6–11 weeks) (*n* = 23). For immunohistochemistry, immunofluorescence and in situ hybridisation, tissues were fixed overnight in 4% paraformaldehyde at 4 °C before processing to paraffin blocks. Primary trophoblasts were isolated from washed placental villi.

### Isolation of trophoblasts

First trimester trophoblasts (*n* = 17) were isolated by enzymatic digestion with trypsin/dispase (Gibco, UK/Roche, Germany), Percoll (Sigma, USA) centrifugation and negative magnetic bead (Dynabeads, Thermo Fisher, Norway) immunopurification with the anti-leukocyte marker CD45 (Invitrogen, Norway) and anti-fibroblast marker CD90 (Dianova, Germany), as described (Blaschitz et al. 2000). Isolated cells were resuspended in DMEM (Gibco) supplemented with 10% FBS. Term trophoblasts (n = 10) were isolated from third trimester placenta as described (Cervar et al. 1999). Briefly, minced villous tissue was digested with a trypsin/dispase/DNase (Sigma) solution. After Percoll-gradient centrifugation a negative selection with magnetic beads conjugated to MCA-81 (Sigma) was performed. Isolated villous trophoblasts were cultured in DMEM supplemented with 10% FBS. First trimester and term trophoblasts were tested for viability and differentiation by measuring β-human chorionic gonadotropin secretion (Dade Behring, USA) (Polliotti et al. 1995). Purity was confirmed by immunocytochemical staining for the trophoblast marker cytokeratin 7 (CK7, Dako, Denmark) and for the mesenchymal cell marker vimentin (Dako) (Cervar et al. 1999). Only preparations of ≥99% purity were used. For gene expression analysis, first trimester (*n* = 10) and term trophoblasts (*n* = 10) were cultured at 37 °C and 21% oxygen for 24 h. For Immunoblotting, first trimester trophoblasts (*n* = 7) were cultured at 37 °C and 5, 12 and 21% oxygen for 48 h.

Primary trophoblasts isolated either from first trimester placenta from week 7 to 10 of pregnancy (GEO accession No GSE59126) or from term placenta (GEO Accession No GSE69086) were subjected to gene expression analysis using HU-133A GeneChips (Affymetrix, Santa Clara, CA). RNA was isolated with Trizol (MRC, Cincinnati, OH, USA) followed by quality assessment using a bioanalyser (Agilent, Palo Alto, CA, USA). For first trimester trophoblasts as well as for term trophoblast, 3 microarrays were performed. On each microarray, total RNA from 5 cell preparations (first trimester trophoblast: GEO Accession Number GSE59126; term trophoblast: GEO Accession Number GSE69086), isolated from different placentas, was pooled. cDNA was synthesised from 5 μg of pooled RNA (SuperScript Double-Stranded cDNA Synthesis Kit; Invitrogen, Carlsbad, USA), transcribed in vitro (RNA Transcript Labeling Kit; Enzo diagnostics, Farmingdale, NY, USA) and fragmented. To test the quality of the cRNA, it was hybridised against test-3 arrays (Affymetrix). As samples passed the quality criteria (bioC, bioD and cre were present, the 3′:5′ ratio of the polyA controls was < 3), the cRNAs were hybridised against Affymetrix HU133A chips. RNA preparation and hybridisation followed the Affymetrix user manual. Data analysis of raw data was normalised globally and processed with Microarray Suite, version 5.0 (Affymetrix) and Data Mining Tool (Affymetrix) software (Huppertz et al. 2009). Genes were classified as being expressed if signal intensity >200. Annotations were obtained from NetAffx (available at http://www.affymetrix.com, last accessed in December 2013).

For protease analysis, we defined proteases as potentially invasion relevant if they cleaved either decidual ECM components (collagens I, III, IV, V, VI, laminins, fibronectin, vitronectin) or trophoblast integrins (integrins α6β4, α5β1 and αvβ3). Using MEROPS database (http://merops.sanger.ac.uk/, release 11.0 (2017)) (Rawlings et al. 2016) we generated a list of 53 proteases capable of cleaving these substrates, of which 46 were analysed by the microarray.

### Semi-quantitative RT-PCR

Total RNA was isolated from isolated trophoblasts from first trimester (*n* = 5) and term placenta (*n* = 5) using the TRI reagent RT kit (Molecular Research Center Inc., Cincinnati, OH, USA) according to the manufacturer’s instructions. Quality and quantity of the RNA were proven utilising the NanoDrop ND-1000 spectrophotometer (PEQLAB Biotechnology GmbH, Erlangen, Germany) and ethidium bromide stained 1% agarose gel electrophoresis. For reverse transcription and amplification, the Qiagen one-step PCR kit (Qiagen, Hilden, Germany) was used on a PTC-200 Thermocycler (GMI Inc., Ramsey, MI, USA) using the preinstalled RT27 program, which consists of three steps starting with the reverse transcription at 50 °C (30 min) and an initial PCR activation step at 95 °C (15 min). The second step includes 27 cycles of denaturation at 94 °C (30 s), annealing at 60 °C (30 s) and extension with a temperature of 72 °C (60 s) followed by the final extension at 72 °C (10 min) in the third step. The gene encoding ribosomal protein L30 (*RPL30*) was used as a housekeeping gene. The primer pair CACCTGACATGAACCGTGA/GCAGAGAGGCGAAATGTGT was used for amplification of *MMP12* and CCTAAGGCAGGAAGATGGTG/CAGTCTGTTCTGGCATGCTT for *RPL30*. Quality assessment was performed on an ethidium bromide stained 1.5% agarose gel, visualised and documented by the ChemiDoc XRS transluminometer (Bio-Rad, Germany).

### Quantitative RT-PCR

Complementary DNA (cDNA) was synthesised with the High Capacity cDNA Reverse Transcription Kit (AB Life Tech Austria, Vienna) using random hexamer primer with the following protocol: 10 min at 25 °C, 120 min at 37 °C for reverse transcription and a 5-s step at 85 °C for enzyme inactivation. The cDNA was diluted 1:20 with nuclease-free water before performing the PCR. For the mastermix 4 µl of 5 ng/µl diluted cDNA, 7.5 µl iQ SybrGreen Supermix (Bio-Rad), 0.2 µM forward and reverse primer was used and filled up to a total volume of 15 µl with nuclease-free water. All samples were run in triplicate. Expression of *MMP12* (forward primer CCACTGCTTCTGGAGCTCTT, reverse primer TCTCGTGAACAGCAGTGAGG) and of the house keeping gene *HPRT1* (forward primer GACCAGTCAACAGGGGACAT, reverse primer CTGCATTGTTTTGCCAGTGT) was measured with CFX96 from BioRad under the following conditions: 95 °C for 3 min, followed by 40 cycles of 95 °C for 10 s, 60 °C for 30 s and 72 °C for 10 s. This was ensued by 30 s at 95 °C and 30 s at 55 °C followed by a melt curve analysis from 55 to 95 °C in 0.5 °C steps lasting 5 s. Data were normalised to *HPRT1* and relative gene expression was assessed using the 2^−ΔΔCt^-method.

### Radioactive in situ hybridisation

Riboprobes were synthesised by in vitro transcription of linearized pDrive plasmid templates containing the appropriate insert, using T7 or SP6 RNA polymerase (Promega) and ^35^S-labelled UTP (NEN/Perkin Elmer) to generate antisense and sense probes. Tissues sections were deparaffinized, fixed in 4% paraformaldehyde and treated with proteinase K. After washing in 0.5 × SSC, the sections were covered with hybridisation solution (50% deionised formamide, 0.3 M NaCl, 20 mM Tris (pH 8.0), 5.0 mM EDTA, 1× Denhardt’s solution, 10% Dextran Sulphate, and 10 mM DTT) and prehybridised for 2 h at 55 °C. ^35^S-labelled antisense and sense RNA probes (3 × 10^5^ cpm/slides) were added to the hybridisation solution, and the incubation continued for 12–18 h at 57 °C. After hybridisation, the sections were washed for 20 min in 2X SSC, 10 mM β-mercaptoethanol and 1 mM EDTA, treated with RNAse A (10 µg/ml) for 30 min at room temperature and washed at high stringency (0.1 x SSC, 10 mM β -mercaptoethanol, 1 mM EDTA) for 2 h at 60 °C; 20 min in 0.5 × SSC. Tissue sections were then dehydrated in graded ethanol containing 0.3 M ammonium acetate, air dried, and dipped in photographic emulsion NTB-2 (Kodak). Slides were stored at 4 °C in light-tight boxes; after 6 weeks’ exposure sections were developed and counterstained with haematoxylin and eosin.

### DIG in situ hybridisation

Digoxigenin-11-UTP Labeling Kit with T7 and SP6 RNA polymerases was used (Roche Diagnostics Corp. Indianapolis, USA) to generate antisense and sense probes. Briefly, tissue sections were deparaffinised, fixed in 4% paraformaldehyde and treated with proteinase K. After washing in 0.5 × SSC, the sections were covered with 100 ml of hybridisation solution (50% deionized formamide, 0.3 M NaCl, 20 mM Tris (pH 8.0), 5.0 mM EDTA, 1× Denhardt’s solution, 10% Dextran sulfate, and 10 mM DTT) and prehybridised for 30 min at 55 °C. Excess liquid was drained off before 100 µl of probe was added and the incubation continued for 12–18 h at 57 °C. After hybridisation, the sections were washed for 20 min in 2 × SSC and 1 mM EDTA, treated with RNAse A (10 µg/ml) for 30 min at room temperature and washed at high stringency (0.1 x SSC, 1 mM EDTA) for 2 h at 60 °C; 20 min in 0.5 × SSC. Hybridised probe was detected using an Anti-Digoxigenin-AP, Fab fragments (Roche and NBT-BCIP colour substrate mix (Roche). Sections were counter stained with 0.1% (w/v) methyl green (Sigma).

### Immunofluorescence

Studies were carried out on 4% PFA-fixed, paraffin-embedded placental villous and decidual tissues of first trimester and term placenta, sectioned at 5 µm. Slides were deparaffinised in xylene and rehydrated with decreasing concentrations of ethanol according to standard methods. Antigen retrieval was used for tissue sections submerged in 10 mM Sodium Citrate Buffer, 0.05% Tween-20 (pH 6.0) for 10 min in a domestic microwave oven. Slides were allowed to cool down for 45 min at room temperature before rinsing in wash buffer Tris-Buffered Saline Tween-20 (TBST, pH 7.4). Sections were blocked with UV ultra block for 7 min before primary antibody incubation at 4 °C overnight. In all double stainings, rabbit anti-human MMP-12 antibody was used to stain MMP12 (0.67 µg/ml, ab38935, Abcam, Cambridge, MA). Sections were costained for HLA-G, a marker for extravillous trophoblasts (mouse anti human HLA-G clone MEM-G/1, 0.5 µg/ml, BioVendor, Heidelberg, Germany), for cytokeratin 7, a marker for all trophoblast subpopulations (mouse anti-human cytokeratin-7 clone PG-M1, 0.21 µg/ml, Dako Cytomation) and for Ki67, a marker for proliferating cells (mouse anti human Ki67 clone MIB-1, 1.07 µg/ml, Dako Cytomation). Negative controls were incubated with the appropriate IgG fractions as isotype controls. All incubation steps were performed in a dark moist chamber at room temperature. After 5 min of TBST wash, secondary antibodies (goat anti-rabbit labelled with Dylight488 and goat anti-mouse conjugated with Cy3, diluted 1:800, Jackson ImmunoResearch Laboratories, West Grove, PA) were applied for 30 min. Followed by another TBST wash, DAPI (blue) 5 mg/ml was added to the slides for 20 min as a nuclei counter stain. The sections were rinsed again with TBST before mounting with Vectashield mounting medium (Vector Lab, Inc., Burlingame, CA, USA). Images were acquired on a Leica DM4000 B trinocular microscope equipped with a Leica DFC300 FX digital camera and processed with the image capturing software Leica Application Suit, Version 3.6.0 (all three: Leica Microsystems Ltd., Heerbrugg, Switzerland).

### Quantification of MMP12 positive cells

HLA-G positive cells and MMP12 positive cells within the cell columns were quantified. Only cell columns that were entirely cross sectioned (from the villus to the distal part of the column) on the slide were used for analysis and tagged using GIMP (Open Source, GNU General Public License, Version 3) and subsequently counted by Motic Images Advanced 3.2 (Motic Deutschland GmbH, Germany). Data were analysed in Microsoft Excel (Microsoft, Redmond, WA) by calculating the ratio of MMP12 positive cells among the HLA-G positive cells for each cell column.

### Promoter analysis

The *MMP12* promoter was analysed for potential transcription factor binding sites using PROMO bioinformatics tool (Version 3.0.2) at http://alggen.lsi.upc.es/cgi-bin/promo_v3/promo/promoinit.cgi?dirDB=TF_8.3. *MMP12* promoter sequence (2000 bp upstream of the transcriptional) was obtained from UCSC Genome Browser (https://genome.ucsc.edu/). The maximum matrix dissimilarity rate was restricted to 2% and the Random Expectation query, which gives the number of expected occurrences of the match in a random sequence, was restricted to <0.2.

### Immunoblotting

Immediately after isolation, primary first trimester trophoblasts from 4 different placentas, each in triplicate, were cultured at 5, 12 and 21% oxygen for 48 h. With an additional set of experiments, a further treatment with the HIF-1α activator Desferrioxamine (DFO, Sigma) was performed at 21% oxygen. DFO is an iron chelator that prevents HIF-1α degradation and thus, promotes HIF-1 action (Salceda and Caro 1997). To test the optimal DFO concentration, 10, 50 and 100 µM DFO was added. Since 100 µM gave the best results, this concentration was chosen for the experiments. DFO treatment was performed in primary first trimester trophoblasts from three different placentas, each in triplicate. For Western blotting, lysates of total cell proteins were prepared in RIPA buffer (Sigma) containing protease inhibitors (Roche). Protein concentration was determined by the bicinchoninic acid assay (BCA). Equal amounts of protein were mixed with Laemmli buffer (Sigma) and denatured at 96 °C for 5 min. Samples were loaded onto 10% SDS-PAGE gels (Bio-Rad), resolved at 140 V for 1 h and transferred to nitrocellulose membranes (Bio-Rad). Non-specific binding sites were blocked for 1 h with 5% non-fat dry milk (Bio-Rad) in tris-buffered saline (TBS) +0.1% Tween 20 (Sigma). After blocking, membranes were incubated with anti MMP12 antibody (Abcam, 1:1000) and or β-actin (Abcam, 1:25,000) overnight at 4 °C. Blots were subsequently washed and incubated with the HRP-conjugated secondary antibody (goat anti rabbit, Bio-Rad Laboratories, 1:2000 for MMP12 and 1:25,000 for β-actin). Immunolabeling was detected using SuperSignal West Pico Chemiluminescent Substrate (Thermo Scientific) and visualised with the Chemidoc XRS software (Bio-Rad). Band density was quantified using the Alpha Digidoc software (Alpha Innotech Corp, Innsbruck, Austria).

### Statistical evaluation

Kruskal–Wallis test was used to compare the ratio of MMP12 positive cells in the different gestational weeks and to analyse the oxygen effect on MMP12 levels and for pairwise analysis, Dunn’s Multiple Comparison Test as post test for pairwise analysis.

## Results

### MMP12 expression is specific for first trimester trophoblasts

**Fig. 1:**
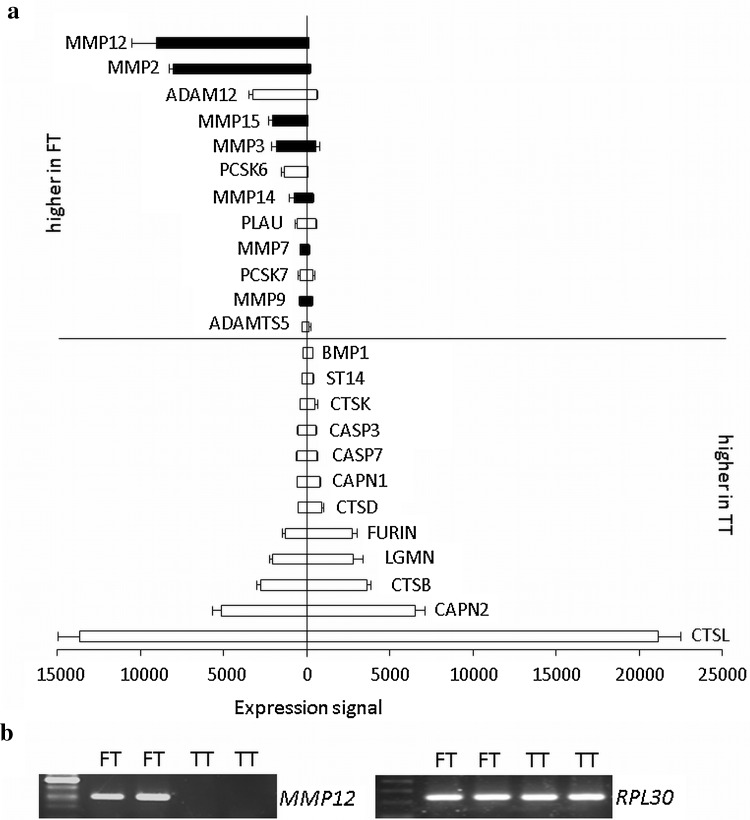
(**a**) Proteases capable to degrade decidual ECM and integrins, expressed by primary trophoblasts from first trimester (FT) and term of gestation (TT). Among all proteases degrading collagens I, III, IV, V and VI, laminin, fibronectin, fibrillin 1 and 2, vitronectin and integrins specific for extravillous trophoblasts, *MMP12* was highest expressed in FT with almost absent expression in TT. MMPs are coloured in black. Data are shown as mean ± SD. **b** The expression difference of *MMP12* in primary first trimester (FT) vs. term trophoblasts (TT) was confirmed by semiquantitative RT-PCR using *RPL30* as housekeeping gene

In order to identify proteases specifically important for trophoblast invasion, we compared gene expression of potentially invasion relevant proteases between primary FT, representing an invasive phenotype paralleled by expression of HLA-G (Ghaffari-Tabrizi-Wizsy et al. 2014), with TT, representing a non-invasive phenotype. Proteases were selected according to their ability to degrade major ECM components of the decidua, i.e. collagens I, III, IV, V and VI, laminin, fibronectin, fibrillin 1 and 2 and vitronectin (Kemp et al. 2002; Oefner et al. 2015; Aplin et al. 2000). Furthermore, we included proteases capable to cleave integrins expressed by invasive trophoblasts, i.e. integrins α6β4, α5β1 and αvβ3 (Kemp et al. 2002). Using MEROPS database, we identified 53 human proteases involved in cleavage of these substrates, of which 46 were measured by the microarray (Online Resource 1). 24 of these proteases were expressed in FT with 12 of them being higher expressed in FT than in non-invasive TT. These were regarded as potentially invasion relevant. The proteases with highest expression level in FT and with largest expression difference (84-fold) between FT and TT were *MMP12* (Fig. 1a). Gene expression of *MMP12* was confirmed by semi-quantitative RT-PCR (Fig. 1b) and by quantitative RT-PCR (not shown). Besides MMP12, also other MMPs (MMP2, 15, 3, 14, 7 and 9) and ADAMs (ADAM12 and ADAMTS5) were identified by the screening, as well as PLAU (urokinase-type plasminogen activator) and the two serine proteases PCSK6 and 7 (proprotein convertase subtilisin/kexin type-6 and -7).

### MMP12 expression is specific for evCT

**Fig. 2:**
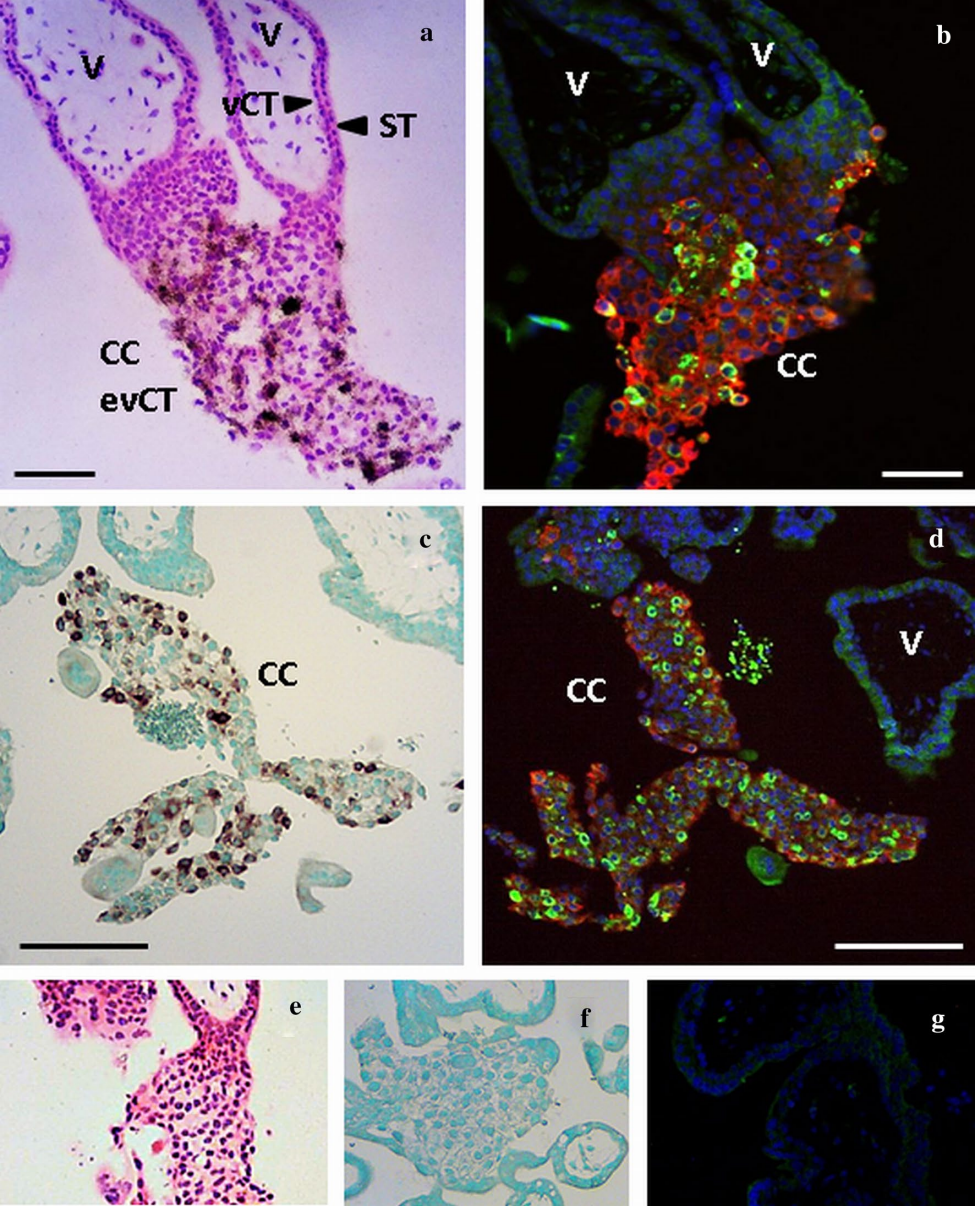
Localisation of MMP12 mRNA and protein in the first trimester placenta with radioactive (**a**) and DIG labelled (**c**) in situ hybridisation and immunofluorescence (**b**, **d**). Cells stained positive for *MMP12* mRNA give a black (**a**) or brown (**c**) signal in the in situ hybridisation methods. Immunofluorescence costained MMP12 (green) with HLA-G (red). **a** and **b**, **c** and **d** represents serial sections. **a**–**d** MMP12 is located in the trophoblasts of the cell columns (CC) and absent in the placental villi (V). *ST* syncytiotrophoblast, *vCT* villous cytotrophoblast, *evCT* extravillous cytotrophoblasts. Original magnification: ×100. Size of the scale bars: 100 µm (**a**, **b**) or 200 µm (**c**, **d**). Negative controls for the radioactive (**e**), DIG labelled (**f**) and immunofluorescence (**g**) stainings

**Fig. 3:**
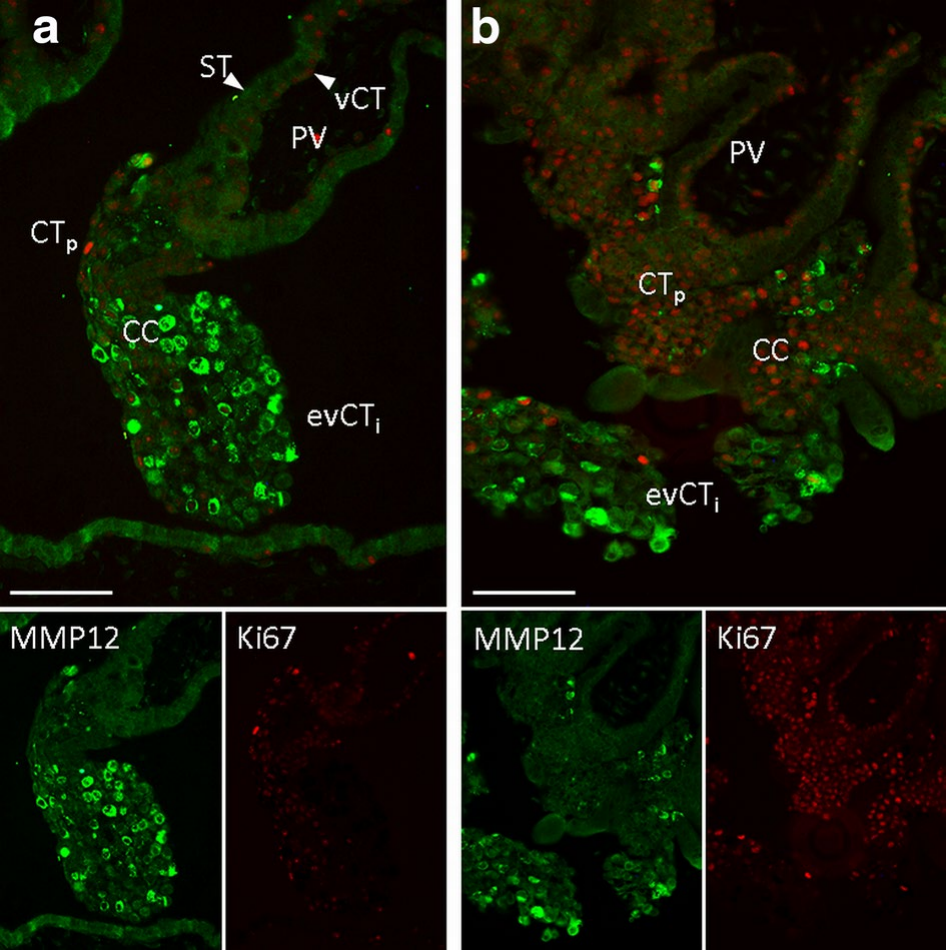
Colocalisation of MMP12 (green) and the proliferation marker Ki67 (red) in the cell columns of the first trimester placenta using immunofluorescence (**a**, **b**). Ki67 antibody stained proliferating villous cytotrophoblasts (vCT) and the proliferative phenotype of the proximal part of the cell columns (CT_p_). MMP12 antibody predominantly stained the invasive of extravillous cytotrophoblasts (evCT_i_). **a** and **b** are representative images of *n* = 6 different placentas. The green (MMP12) and red (Ki67) channel images are shown separately in the inserts below the overlay. *ST* syncytiotrophoblast, *PV* placental villus, *CC* cell column. Original magnification: ×200. Size of the scale bars: 100 µm

Localisation of MMP12 mRNA and protein in first trimester placental tissues using in situ hybridisation and immunofluorescence confirmed the potential role of MMP12 in trophoblast invasion. In situ hybridisation was performed using two different techniques, i.e. radioactive vs DIG labelled probes. For immunofluorescence, sections were double stained for HLA-G, a marker for evCT (Moser et al. 2011). All methods revealed positive MMP12 mRNA and protein expression in a specific subset of the evCT of the cell columns The MMP12 antibody revealed also a faint signal in the syncytiotrophoblast, in villous cytotrophoblasts and in some stromal cells. However, since both in situ hybridisation methods gave no signal for *MMP12* mRNA in these compartments, limited production of MMP12 protein by these cells may be possible but precarious (Fig. 2a–d). Moreover, as described previously (Harris et al. 2010), MMP12 was present in the evCT invading and remodelling the uterine vessels, i.e. endovascular trophoblasts (not shown). Within the cell columns, MMP12 expression was primarily located to a distinct, scattered subpopulation. In order to identify whether MMP12 production may be specific for proliferating cells, sections were co-stained for the proliferation marker Ki67. The proliferative trophoblasts of the proximal part of the cell columns were stained for Ki67, but only few cells were positive for MMP12, whilst the invasive evCT of the distal part of the columns produced MMP12, but were mostly negative for Ki67 (Fig. 3a, b).

### Temporal changes in expression of MMP12 by evCT

**Fig. 4:**
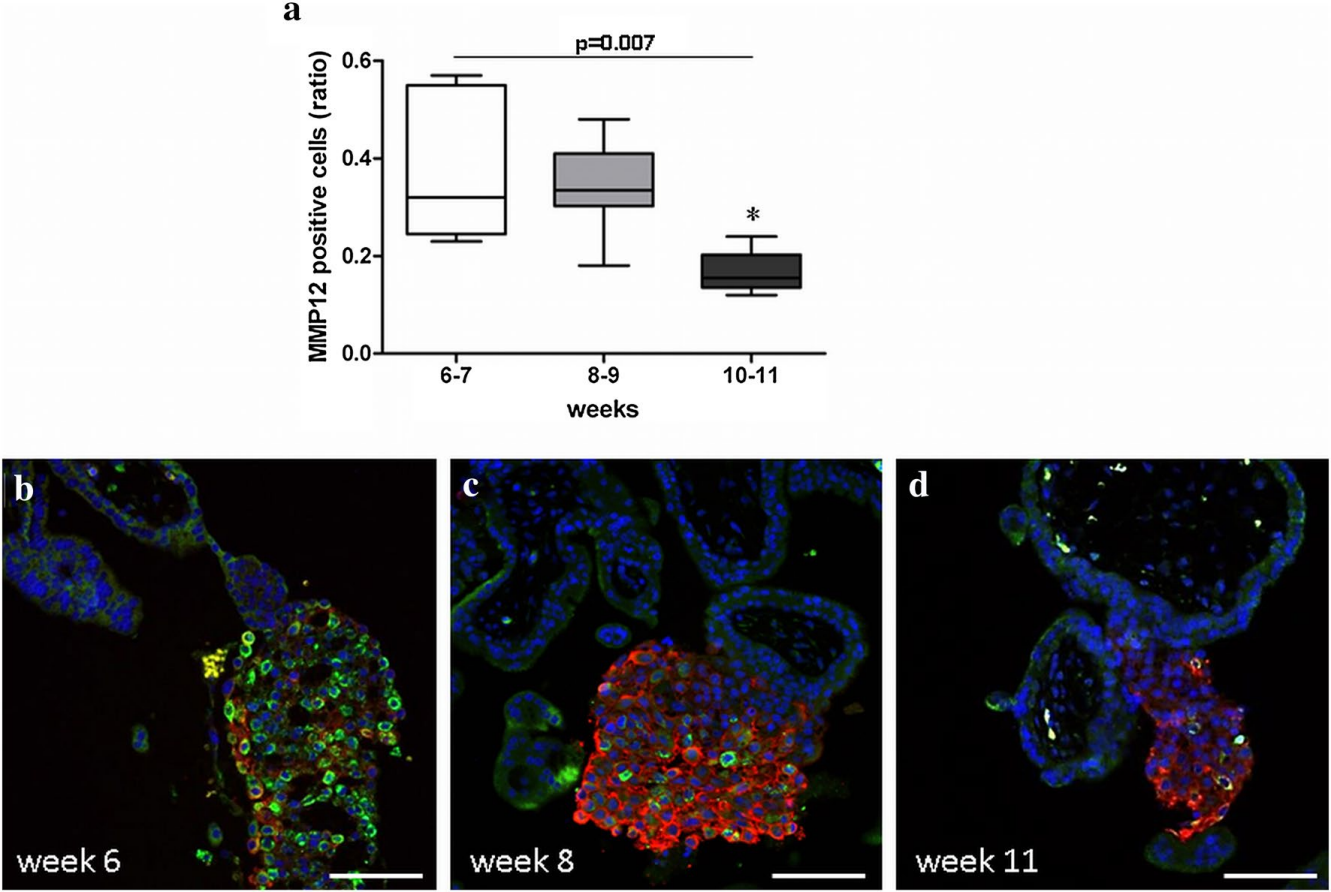
Ratio of MMP12 positive cells (green) within HLA-G positive extravillous trophoblasts (red) of the cell columns throughout the first trimester of pregnancy (**a**). *n*_(6–7)_ = 5, *n*_(8–9)_ = 8, *n*_(10–11)_ = 6 different tissues. Of each tissue, three sections were analysed. Statistical analysis used non-parametric ANOVA (Kruskal–Wallis). Post test (Dunn’s Multiple Comparison) revealed a significant reduction in the ratio of MMP12 positive cells in weeks 10–11 (asterisk). The box plot indicates the median, 25th and 75th percentile; whiskers show minimum and maximum values. **b**–**d** Representative images of each group are shown below. Original magnification: ×100. Size of the scale bars: 100 µm

In order to determine whether MMP12 expression during the first trimester is temporally regulated, we quantified MMP12 positive cells in placental specimen of different gestational weeks, i.e. in weeks 6–7, 8–9 and 10–11, using image analysis of HLA-G and MMP12 double-labelled cells (Online Resource 2). There was a significant reduction in the ratio of MMP12 positive cells within all evCT towards the end of the first trimester, i.e. in weeks 10–11 vs earlier weeks (Fig. 4).

### MMP12 expression is regulated by oxygen

**Fig. 5:**
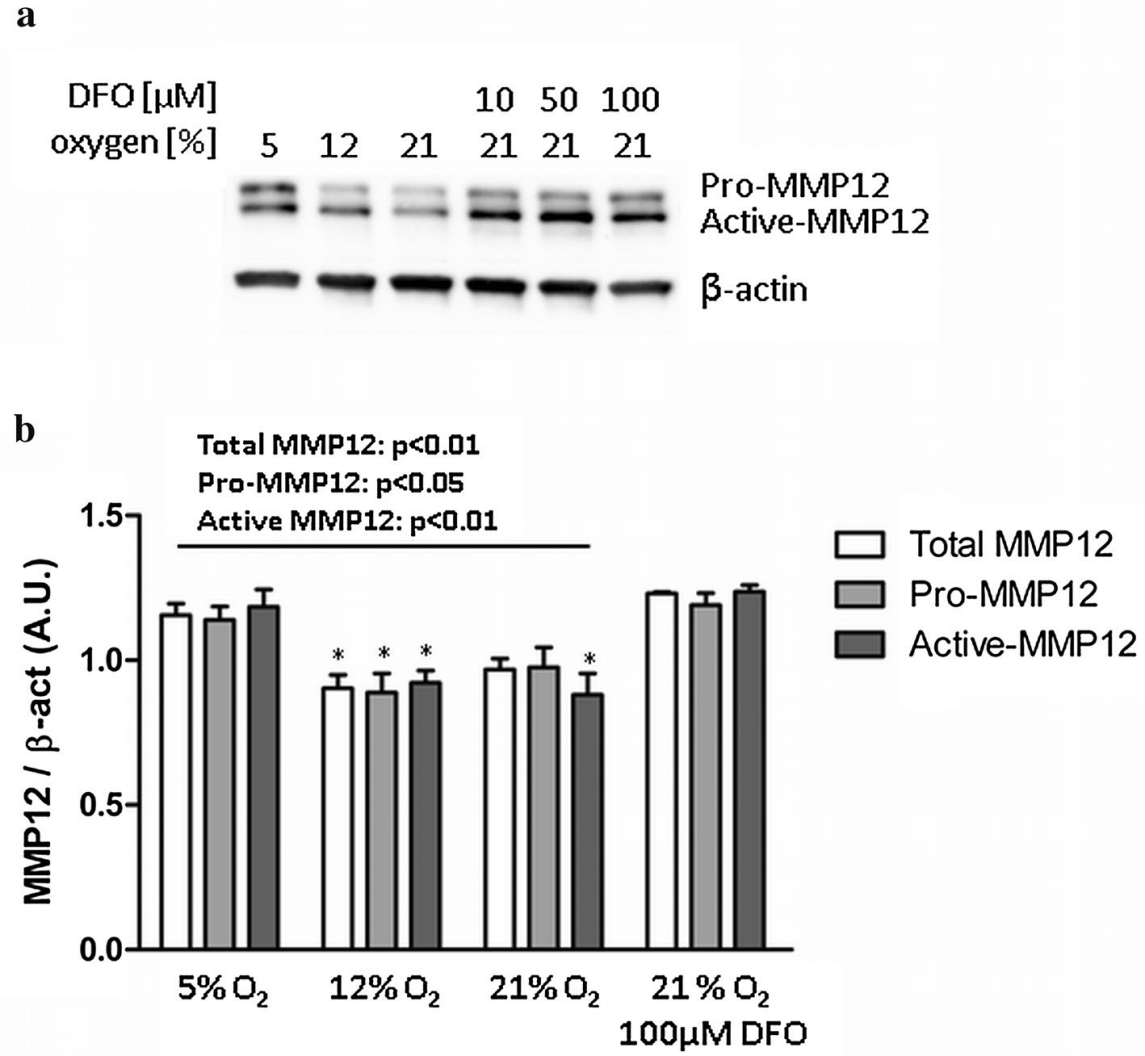
Effect of oxygen on MMP12 protein in primary first trimester trophoblasts. Isolated trophoblasts were cultured at 5, 12 and 21% oxygen for 48 h. A representative immunoblot is shown on top (**a**). Quantified data are given as mean ± SEM (**b**). *n* = 7 different trophoblast isolations between weeks 7 and 11, each in duplicate. Statistical analysis used non-parametric ANOVA (Kruskal–Wallis). Post test (Dunn’s Multiple Comparison) revealed a significant reduction in MMP12 protein at 12 and 21% oxygen when compared to 5% oxygen (asterisk), but oxygen effect was abrogated when the HIF-1α activator DFO was added

To determine potential regulators of *MMP12* expression, a transcription factor binding site (TFBS) analysis of the *MMP12* promoter using PROMO bioinformatics tool (Messeguer et al. 2002; Farre et al. 2003; Fekry et al. 2016) was performed. The analysis revealed TFBS already described in the *MMP12* promoter, such as AP-1, wnt signalling and PEA3 binding sites, but in addition furthermore identified other potential TFBS, including a HIF-1 binding site (Online Resource 3). Thus, several binding sites for hypoxia-sensitive transcription factors exist in the *MMP12* promoter, including HIF-1. Therefore, we investigated the impact of oxygen on MMP12 regulation and measured MMP12 protein in primary FT cultured at different oxygen concentrations. Immunoblotting demonstrated that higher levels of oxygen significantly reduced MMP12 when compared to 5% oxygen (Fig. 5). Concordantly, activation of HIF-1α with its specific activator DFO increased MMP12 level even in the presence of 21% oxygen (Fig. 5).

## Discussion

Trophoblast invasion depends on proteases. We here aimed to identify proteases potentially relevant for this fundamental process in pregnancy establishment. Gene expression analysis identified MMP12 as a proteolytic enzyme highly expressed in primary FT that might be capable of degrading decidual ECM. Both, in situ hybridisation and immunofluorescence, localised MMP12 mRNA and protein in vivo to evCT at the distal part of the cell columns and showed that the number of these MMP12 producing cells declined towards the end of the first trimester. Moreover, we have demonstrated that MMP12 is upregulated under low oxygen and that HIF-1α activity induces MMP12 production, suggesting that the rise in oxygen at the end of the first trimester may account for this temporal regulation of MMP12.

In order to analyse the location of MMP12 we used several techniques to detect mRNA and protein in situ. Data revealed that MMP12 was expressed in a distinct evCT population, whilst it was absent in the villous cytotrophoblasts and in the syncytiotrophoblast. Concordant with previous literature, we also detected MMP12 in endovascular trophoblasts where it was previously proposed as key player in endovascular remodelling of spiral arteries due to its elastolytic activity (Harris et al. 2010). In contrast to other reports showing MMP12 mRNA or protein expression either in all trophoblast subpopulations, including villous cytotrophoblasts and syncytiotrophoblast (Harris et al. 2010), or homogeneously distributed in all trophoblasts in the cell columns (Chakraborty et al. 2016), we located MMP12 mRNA and protein expression predominantly to non-proliferating, highly invasive evCT of the distal part of the cell columns, whilst the proliferating trophoblasts of the proximal cell columns were mostly negative for MMP12.

Since MMP12 is capable of degrading various substrates present in the decidua including collagen III, fibrillin, fibronectin and laminin (Rawlings et al. 2016), MMP12 may promote decidual ECM breakdown for the invasion of evCT. Moreover, MMP12 is a potent converter of plasminogen into angiostatin (Cornelius et al. 1998). Angiostatin is an antiangiogenic molecule, has anti-proliferative effects on endothelial cells (Brauer et al. 2011) and was also shown to reduce MMP production in JEG-3 trophoblast model cells (Zhang et al. 2003). Plasmin is an alternative cleavage product of plasminogen by the plasminogen activator PLAU. Plasmin is a protease by itself, activates various MMPs (Carmeliet et al. 1997) and degrades ECM components, thus promoting invasion. Therefore, cleavage of plasmin into angiostatin by MMP12 promotes the non-invasive role of the plasminogen gene product and may represent a feedback loop to limit trophoblast MMP production, and hence, invasiveness. Thus, although a role of MMP12 in trophoblast invasion was not directly shown, its ability to degrade ECM degrading enzyme presumes such a function.

Trophoblast invasion is highest in the first trimester and declines thereafter (Hohn and Denker 2002; Shimonovitz et al. 1994). Concordantly, trophoblast production of MMPs is high during the first gestational weeks and is often attenuated already during, or after the first trimester (Xu et al. 2000). Oxygen is a key regulator of this temporal reduction of trophoblast invasion: Before the opening of uterine spiral arteries, foeto-placental development occurs in a low-oxygen environment that is thought to protect the foetus from oxidative stress. However, when maternal blood flow to the placenta starts at the beginning of the second trimester, the foeto-placental oxygen levels rise as well. In fact, low oxygen is considered as a main stimulator of trophoblast invasiveness (James et al. 2006) and the expression of various proteases lowers after the increase in placental oxygenation. For instance, some MMPs were shown to be regulated directly by HIF-1α, such as MMP1, MMP3 (Lin et al. 2008) and MMP14 (Wan et al. 2011).

Also the regulation of MMP12 by oxygen suggested a role of HIF-1α as a classical mediator of oxygen effects. In fact, promoter analysis identified a potential HIF-1 binding site in the *MMP12* promoter (Supplemental Fig. 2) and addition of DFO, a HIF-1α activator, upregulated MMP12 protein. However, the effect of HIF-1 in stimulating MMP12 levels may also be mediated by wnt signalling, since β-catenin as the central molecule of wnt signalling is a target gene of HIF-1 (Liu et al. 2010) and the *MMP12* promoter contains a LEF1/TCF4 binding site for wnt-induced transactivation. Moreover, the LEF1/TCF4 binding site in the *MMP12* promoter provides a mechanism for gene transactivation, specifically in the non-proliferating evCT (Pollheimer et al. 2006). Transactivation of MMP12 by direct and indirect HIF-1 effects, however, may be further enhanced by activator protein 1 (AP-1) which has two binding sites in the *MMP12* promoter (Yan and Boyd 2007) and can also be activated by oxidative stress (Webster et al. 1993). A potential binding site was also found for upstream transcription factor 2 (USF2), a transcription factor that was shown to regulate hypoxia responsive genes in trophoblasts (James et al. 2006). Finally, Chakraborty et al. (2016) demonstrated hypoxia-induced *MMP12* expression in trophoblasts isolated from second trimester placenta (weeks 20–22), but did not assign this response to a direct effect of HIF-1, but to histone demethylation by lysyl demethylase 3A (KDM3A), which is under HIF-1 control (Wellmann et al. 2008). Indeed both, histone demethylation by KDM3A and binding of HIF-1 to its consensus sequences, may cooperate in transcriptional activation of their target genes (Mimura et al. 2012). Thus, various promoter elements in the MMP12 promoter enable MMP12 transactivation by changes in oxygen concentrations and by HIF-1 in particular, highlighting the role of oxygen in regulating MMP12 expression. Which of these possible scenarios account for our observed HIF-1 effect can only be speculated here and would require further, more detailed studies, but interplay of several pathways is likely.

In screening for invasion-relevant proteases we focused on proteases capable of degrading decidual ECM and trophoblast integrins. Proteases may also regulate activation and degradation of cytokines, growth factors and other proteases, and modulate trophoblasts in that way. Therefore, also other proteases aside from what we have selected will modulate trophoblast invasion; however, our main focus was on proteases that directly target ECM degradation.

Our screening approach to identify invasion-relevant proteases revealed *MMP12* expression levels in FT even higher than levels of *MMP2* and *MMP9*, the two most investigated proteases in trophoblast invasion, which were also identified as invasion relevant by our screening. Moreover, we also found several other MMPs and ADAMs, highlighting the role of these two metalloprotease families in pregnancy establishment. Also PLAU has earlier been implicated in invading trophoblast invasion (Anteby et al. 2004). Two proteases, however, have not yet been assigned to trophoblast invasion, i.e. PCSK6 and PCSK7. Both enzymes possess a broad range of substrates, including integrins, fibrillin and various MMPs and ADAMs (MEROPS database). In fact, PCSK6 was implicated in cancer invasion (Wang et al. 2015), a process that resembles trophoblast invasion in many instances.

In this study, we used primary FT and TT as models for invasive and non-invasive trophoblast phenotypes, respectively. Although these models are primary cells, we are aware that taken out of their physiological environment and cultured in vitro, they are exposed to artificial environment which may alter their functionality or differentiation status. However, cultured FT isolated by our method are HLA-G positive (Ghaffari-Tabrizi-Wizsy et al. 2014) and perform in vitro invasion (Majali-Martinez et al. 2017), whilst cultured TT represent a HLA-G negative trophoblast population able to differentiate and fuse into syncytial structures (Simpson et al. 1992). Therefore, we regard these cells as an appropriate model for our screening approach.

The screening approach for invasion-relevant proteases was limited by the fact that FT were cultured at ambient oxygen levels, as in vivo FT are exposed to low-oxygen environment. However, the experiments measuring the effect of oxygen on MMP12 levels in FT revealed that the distinct expression of MMP12 in FT vs TT by far exceeded this effect of oxygen.

We have shown that MMP12 is specifically expressed by non-proliferating invasive trophoblasts of the trophoblast cell columns and that MMP12 expressing cells decrease at the end of the first trimester. Furthermore, we have demonstrated oxygen as a key regulator for *MMP12* expression and proved HIF-1 to induce *MMP12*. This may occur either directly via the potential HIF-1 binding site in the *MMP12* promoter or indirectly via upregulation of other transcriptional regulators by HIF-1. Therefore, this study implies that MMP12 may represent a key protease in trophoblast invasion during first trimester pregnancy.

## Electronic supplementary material

Below is the link to the electronic supplementary material.

Supplementary material 1 (DOCX 5109 kb)
