# Supplementary material for: Expression of matrix metalloproteinase 12 is highly specific for non-proliferating invasive trophoblasts in the first trimester and temporally regulated by oxygen-dependent mechanisms including HIF-1A
Source: Histochem Cell Biol. 2017 Oct 9;149(1):31–42. doi: 10.1007/s00418-017-1608-y (PMC5767211; doi:10.1007/s00418-017-1608-y)
Supplement: Supplementary file 1 — Supplementary material 1 (DOCX 5109 kb) [file 418_2017_1608_MOESM1_ESM.docx]

**Online Resource 1** Potentially invasion relevant human proteases which cleave decidual ECM components (collagens I, III, IV, V and VI; laminin; fibronectin; fibrillin 1, 2; vitronectin) and trophoblast integrins (α6β4; α5β1; αvβ3) and their substrates. The list was obtained from MEROPS database. Proteases in grey were not covered by the microarray analysis. Proteases in bold were expressed in isolated primary first trimester trophoblasts.

| **Gene name** | **Gene symbol** | **Substrate** |
| --- | --- | --- |
| ADAM8 peptidase | *ADAM8* | FN1 |
| ADAM12 peptidase | ***ADAM12*** | COL IV, FN1 |
| procollagen I N-peptidase | *ADAMTS2* | COL I |
| ADAMTS4 peptidase | *ADAMTS4* | COL I |
| ADAMTS5 peptidase | ***ADAMTS5*** | COL III, FN1 |
| procollagen C-peptidase | ***BMP1*** | COL I, LAMG2 |
| calpain 1 | ***CAPN1*** | ITGB1, ITGB3 |
| calpain 2 | ***CAPN2*** | ITGB1, ITGB3 |
| caspase 3 | ***CASP3*** | ITGB4 |
| caspase 7 | ***CASP7*** | ITGB4 |
| chymase | *CMA1* | COL I , FN1 |
| cathepsin B | ***CTSB*** | COL III |
| cathepsin D | ***CTSD*** | COL I, IV |
| cathepsin K | ***CTSK*** | COL I |
| cathepsin L | ***CTSL*** | COL I |
| elastase 2 | *ELANE* | COL III, FN1, LAMA1, LAMB1, LAMG1, LAMG2 |
| furin | ***FURIN*** | ITGA5, ITGA6, FBN1, VTN |
| granzyme B | *GZMB* | VTN |
| hepsin | *HPN* | LAMB3 |
| kallikrein-related peptidase 2 | *KLK2* | FN1 |
| kallikrein-related peptidase 3 | *KLK3* | FN1 |
| kallikrein-related peptidase 4 | *KLK4* | FN1 |
| kallikrein-related peptidase 5 | *KLK5* | VTN |
| kallikrein-related peptidase 8 | *KLK8* | FN1 |
| kallikrein-related peptidase 13 | *KLK13* | FN1, LAMA5 |
| kallikrein-related peptidase 14 | *KLK14* | COL IV, FN1, LAMA5, VTN |
| legumain | ***LGMN*** | COL IV, FN1 |
| meprin alpha subunit | *MEP1A* | FN1, LAMA1 |
| meprin beta subunit | *MEP1B* | FN1, LAMA3 |
| matrix metallopeptidase 1 | *MMP1* | COL I |
| matrix metallopeptidase 2 | ***MMP2*** | COL I, III, IV, V, VI, FBN2, FN1, LAMA1 |
| matrix metallopeptidase 3 | ***MMP3*** | COL IV, FN1, LAMG2 |
| matrix metallopeptidase 7 | ***MMP7*** | COL I, LAMB3, ITGB4 |
| matrix metallopeptidase 8 | *MMP8* | COL I, III, FN1, LAMG2 |
| matrix metallopeptidase 9 | ***MMP9*** | COL IV, FBN1, FBN2, FN1, LAMA1, LAMB1 |
| matrix metallopeptidase 12 | ***MMP12*** | COL I, III, FBN1, FBN2, FN1, LAMG2 |
| matrix metallopeptidase 13 | *MMP13* | COL I, III, FBN1, FBN2, LAMG2 |
| membrane-type matrix metallopeptidase 1 | ***MMP14*** | COL I, ITGAV, LAMA5 |
| membrane-type matrix metallopeptidase 1 | ***MMP15*** | FN1, LAM |
| membrane-type matrix metallopeptidase 3 | *MMP16* | COL III |
| matrix metallopeptidase 20 | *MMP20* | LAMG2 |
| matrix metallopeptidase 26 | *MMP26* | FN1, VTN |
| PCSK2 peptidase | *PCSK2* | FBN1, ITGA5, ITGA6 |
| PCSK4 peptidase | *PCSK4* | FBN1, ITGA5, ITGA6 |
| PCSK5 peptidase | *PCSK5* | FBN1, ITGA5, ITGA6 |
| PCSK6 peptidase | ***PCSK6*** | FBN1, ITGA5, ITGA6 |
| PCSK7 peptidase | ***PCSK7*** | FBN1, ITGA5, ITGA6 |
| pepsin A | *PGA3* | COL I |
| urokinase-type plasminogen activator | ***PLAU*** | ITGA6 |
| plasmin | *PLG* | VTN |
| trypsin 1 | *PRSS1* | COL IV , V, VI, FN1, LAMB2 |
| matriptase | ***ST14*** | FN1, LAMA1 |
| tolloid-like 1 protein | *TLL1* | COL I,III, IV, LAMG2 |


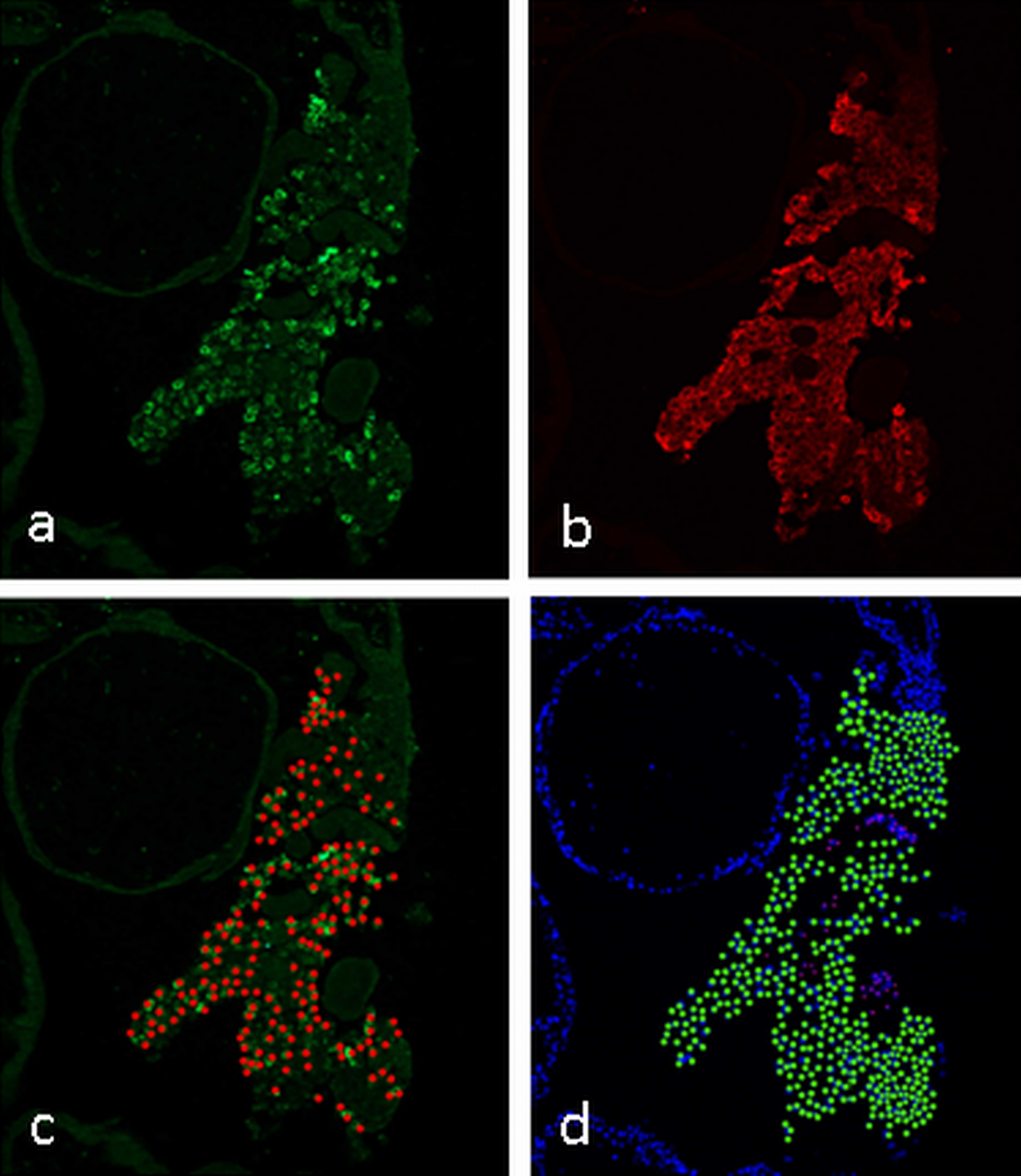


**Online Resource 2** Quantification of MMP12 positive cells (**a**, green fluorescence) within the extravillous trophoblasts of the cell column (HLA-G positive cells, **b**, red fluorescence). MMP12 positive cells were marked with red dots (**c**), and HLA-G positive cells with green dots (**d**) using GIMP software, and automatically counted using Matic Images Advanced 3.2 software.


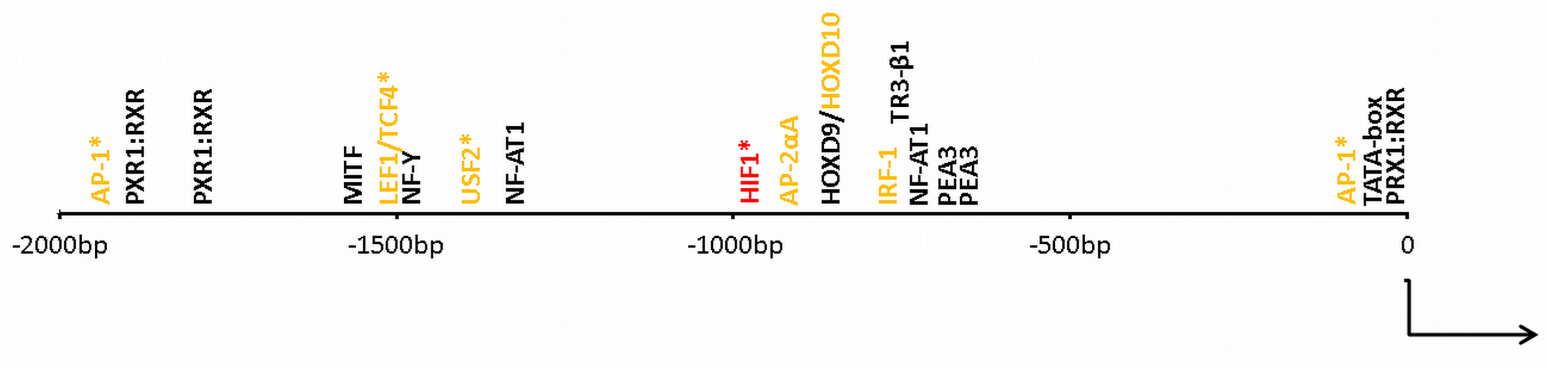


**Online Resource 3** Potential transcription factor binding sites (TFBS) in the *MMP12* promoter. Data were obtained using PROMO 3.0.2 bioinformatics tool (maximum matrix dissimilation = 2, random expectation query value < 0.2). TFBS that were shown to transactivate other MMPs or genes involved in trophoblast invasion are shown in orange (AP-1: activator protein 1, is responsive to cytokines and growth factors and regulates the invasive and endocrine phenotype of trophoblasts (Kubota et al. 2015); LEF1/TCF4: lymphoid enhancer binding factor 1/transcription factor 4, target of wnt signalling with TCF4 almost exclusively expressed in non-proliferating invasive trophoblasts (Pollheimer et al. 2006); USF2: upstream transcription factor 2, induces hypoxia responsive genes in trophoblast (James et al. 2006); HIF-1: hypoxia inducible factor 1, promotes trophoblast invasion (Highet et al. 2015); AP-2αA: transcription factor AP-2 alpha, promotes invasion and upregulates *MMP2* (Biadasiewicz et al. 2011); HOXD9/HOXD10: Homeobox D9/D10: HOXD10 suppresses *MMP14*, *2* and *9* (Liao et al. 2014; Sun et al. 2011), IRF-1: interferon regulatory factor 1, transactivates HLA-G in trophoblasts (Lefebvre et al. 2001)). PXR1:RXR-α: steroid and xenobiotic sensing nuclear receptor; MITF: melanogenesis associated transcription factor; NF-Y: nuclear transcription factor Y; NF-AT1: nuclear factor of activated T-cells; TR3-β1: thyroid hormone receptor. The HIF-1 binding site with the consensus sequence **ACGTGCACC** is highlighted in red. TFBS of transcription factors that may be either induced directly by hypoxia or via HIF-1, are marked with an asterisk.

**References for Online Resource 3**

Biadasiewicz K, Sonderegger S, Haslinger P, et al. (2011) Transcription factor AP-2alpha promotes EGF-dependent invasion of human trophoblast. Endocrinology 152:1458-69. doi: 10.1210/en.2010-0936

Highet AR, Khoda SM, Buckberry S, et al. (2015) Hypoxia induced HIF-1/HIF-2 activity alters trophoblast transcriptional regulation and promotes invasion. Eur J Cell Biol 94:589-602. doi: 10.1016/j.ejcb.2015.10.004

James JL, Stone PR, Chamley LW (2006) The regulation of trophoblast differentiation by oxygen in the first trimester of pregnancy. Hum Reprod Update 12:137-44. doi: 10.1093/humupd/dmi043

Kubota K, Kent LN, Rumi MA, Roby KF, Soares MJ (2015) Dynamic Regulation of AP-1 Transcriptional Complexes Directs Trophoblast Differentiation. Mol Cell Biol 35:3163-77. doi: 10.1128/MCB.00118-15

Lefebvre S, Berrih-Aknin S, Adrian F, et al. (2001) A specific interferon (IFN)-stimulated response element of the distal HLA-G promoter binds IFN-regulatory factor 1 and mediates enhancement of this nonclassical class I gene by IFN-beta. J Biol Chem 276:6133-9. doi: 10.1074/jbc.M008496200

Liao CG, Kong LM, Zhou P, et al. (2014) miR-10b is overexpressed in hepatocellular carcinoma and promotes cell proliferation, migration and invasion through RhoC, uPAR and MMPs. Journal of translational medicine 12:234. doi: 10.1186/s12967-014-0234-x

Pollheimer J, Loregger T, Sonderegger S, et al. (2006) Activation of the canonical wingless/T-cell factor signaling pathway promotes invasive differentiation of human trophoblast. Am J Pathol 168:1134-47. doi: 10.2353/ajpath.2006.050686

Sun L, Yan W, Wang Y, et al. (2011) MicroRNA-10b induces glioma cell invasion by modulating MMP-14 and uPAR expression via HOXD10. Brain Res 1389:9-18. doi: 10.1016/j.brainres.2011.03.013
